# Effects of exercise training at lactate threshold and detraining for 12 weeks on body composition, aerobic performance, and stress related variables in obese women

**DOI:** 10.20463/jenb.2019.0019

**Published:** 2019-09-30

**Authors:** Hun-Young Park, Sungho Kim, Younho Kim, Sangyun Park, Sang-Seok Nam

**Affiliations:** 1. Department of Sports Medicine and Science, Konkuk University, Seoul Republic of Korea; 2. Physical Activity and Performance Institute (PAPI), Konkuk University, Seoul Republic of Korea; 3. Department of Health Rehabilitation, Osan University, Osan Republic of Korea; 4. Samsung Advanced Institute of Technology, Suwon Republic of Korea; 5. Taekwondo Research Institute of Kukkiwon, Seoul Republic of Korea

**Keywords:** obese women, exercise training, detraining, body composition, aerobic performance, stress

## Abstract

**[Purpose]:**

The purpose of this study was to investigate the effect of diet plus exercise training and detraining for 12 weeks on body composition, aerobic performance, and stress-related variables in obese women.

**[Methods]:**

Twenty-five women in their 20s-40s with 30% body fat and body mass indices above 25 kg/m^2^ were divided into HR_LT_ (heart rate at lactate threshold) and HR_LT_ + 5% groups. Dietary intervention of 70% recommended dietary allowance (RDA) and exercise treatment composed of aerobic exercises on a bicycle (30 min) and treadmill (30 min) were then performed. These interventions were performed three times a week for 12 weeks.

**[Results]:**

Dietary intake was significantly decreased, while daily activity significantly increased within the 12-week intervention period, and this effect was sustained after 12 weeks of detraining. Exercise training based on dietary intake and daily activity presented a significantly decreased weight and % body fat, improvement of aerobic performance, and a significant increase in heart rate variability (HRV) (e.g., average of all RR intervals and the square root mean squared differences of successive RR intervals) as stress-related variables. It was also confirmed that the improvement of body composition and stress-related variables were maintained even after detraining.

**[Conclusion]:**

Our results suggest that 70% RDA of dietary intervention and exercise training corresponding to HR_LT_ and HR_LT_ + 5% for 12 weeks were effective in improving body composition and aerobic performance, and relieving stress. In particular, enhanced HRV persisted for up to 12 weeks after the end of exercise training in obese women.

## INTRODUCTION

Obesity is caused by the accumulation of excess fat, an energy imbalance between excess intake of food and calories burned^1^. According to a 2014 report by the World Health Organization (WHO), 39% of the world's adult population (more than 1.9 billion people) are overweight and 13% (6 million) are obese^2^. In Korea, the prevalence of obesity in men decreases with age (32%, 43.9%, 39.6%, 41.5%, 36.9%, and 24.0% in men in their 20s, 30s, 40s, 50s, 60s, and 70s, respectively). However, the prevalence of obesity in women increases rapidly with age (15%, 18.6%, 22.3%, 29.3%, 36.6%, and 37.3% in women in their 20s, 30s, 40s, 50s, 60s, and 70s, respectively)^3^. Hence, there is a greater chance of women becoming obese with increasing age. These results indicate that long-term and continuous intervention for the treatment of obesity is required for women in their 20s to 40s since their prevalence of obesity is relatively low.

Obesity is a major contributory factor to the increasing prevalence of diabetes, hypertension, cardiovascular disease, fatty liver, gallbladder disease, colorectal cancer, and breast cancer; it also causes serious mental, physical, and social problems^4^^,^^5^. Currently, there are three ways to prevent and treat obesity: surgery, medication, and a combination of diet and exercise intervention. Among these, surgery and medication may have an immediate effect on weight loss, but due to the costs involved and associated side effects, they are limited to specific patients such as the morbidly obese in clinical settings^6^^,^^7^. On the other hand, the combination of diet and exercise intervention increases basal metabolic rate by increasing lean body mass and enhancing activation of fat utilization^8^.

In general, the most recommended exercise prescription for the prevention and treatment of obesity is aerobic exercise with moderate intensity (50 to 85% maximal oxygen consumption [VO_2max_] or 60 to 90% maximal heart rate [HR_max_]), frequency (3 to 5 days per week), time (45 to 60 minutes), and duration (more than 12 weeks)^9^^,^^10^. However, the most widely used setting of exercise intensity generally shows different levels of aerobic and anaerobic energy metabolism depending on the VO_2max_ of the individual. Therefore, Menzel & Hilberg^11^ recommend using the anaerobic threshold (AT) intensity of each individual as the appropriate exercise intensity for weight loss. There are two representative methods of determining AT: lactate threshold using blood lactate levels (LT) and ventilatory threshold (VT) using respiratory gas^12^. LT, rather than VT, is more efficient in the determination of AT because it is a point of rapid change in blood lactate concentration, is less subjective in judgment, and more practical^13^.

Recently, the heart rate variability (HRV) test has been widely used in clinical trials as a method for examining the relationship between obesity and the autonomic nervous system^14^^-^^16^. This method is recognized as a non-invasive and reliable evaluation method that can quantitatively evaluate the activity of the sympathetic and parasympathetic nervous systems^17^^,^^18^. Autonomic nervous system function is closely related to obesity; the activity of the sympathetic nervous system leads to a decrease in basal metabolic rate, inducing weight gain, and obesity^19^. Since autonomic nervous system function has been suggested as an important index for evaluating obesity, it is necessary to examine the relationship between the physiological changes caused by weight loss through diet plus exercise intervention and the autonomic nervous system changes following weight recovery after suspending the intervention.

Regular aerobic exercise, composed of appropriate protocols for obese people, has a positive effect on body composition, aerobic exercise capacity, and autonomic nervous system function. In order to maintain these positive changes, steady exercise and a regular lifestyle should be maintained. However, exercise is easily interrupted by such factors as satisfaction with the achievement of the weight goal, daily economic activity, and low interest in exercise. These lead to the yo-yo phenomenon where there is a return to or increase in weight before the weight is lost again^20^. However, studies on the improvement of obesity to date are limited to examining the effects of various types of exercise on various dependent variables including weight loss.

Therefore, our study examined the effect of diet plus exercise training at LT and detraining for 12 weeks on body composition, aerobic performance, and stress-related variables in obese women in their 20s to 40s.

## METHODS

### Participants

The participants of our study were 43 obese women in their 20s-40s with body mass index (BMI) of greater than 25 kg/m2 and 30% body fat. They were divided into two groups made up of 22 women in the exercise group with heart rate at lactate threshold (HR_LT_) and 21 in the HR_LT_ + 5% exercise group by random sampling. Participants underwent 12 weeks of exercise training and detraining consecutively. Of these, 18 participants dropped out due to health problems and personal circumstances during the training period, and five participants who had been inconsistent were excluded during the detraining period. The final number of participants was 25 with 13 women in the HR_LT_ exercise group and 12 in the HR_LT_ + 5% exercise group. All procedures followed were in accordance with the ethical standards of the responsible committee in KyungHee University on human experimentation (KHSIRB 2015-029) and with the tenets of the Declaration of Helsinki. The characteristics of the participants are shown in Table 1.

**Table 1.** Characteristics of participants

Items	HRLT	HRLT + 5%	p-value
Age (yrs)	36.5 ± 10.1	37.2 ± 6.7	.832
Height (cm)	157.4 ± 4.4	161.6 ± 5.4	.116
Weight (kg)	69.1 ± 7.8	71.2 ± 5.4	.451
% BF	34.2 ± 3.8	34.2 ± 2.0	.979
BMI (kg/m^2^)	27.5 ± 2.5	27.3 ± 1.6	.748
THR (bpm)	134.5 ± 14.0	143.2 ± 15.9	.161

Note: % BF = percent of body fat, BMI = body mass index, THR = target heart rate, HRLT = heart rate at lactate threshold.

### Experimental design

In order to conduct our study, we first conducted a graded exercise test (GXT) for the measurement of VO_2max_ on 25 obese women with BMI greater than 25 kg/m^2^ and 30% body fat in their 20s to 40s who had not performed regular physical activity in the previous 6 months. Participants were then divided into two groups of 13 and 12 in the HR_LT_ and HR_LT_ + 5% exercise groups, respectively, by random sampling. All participants underwent training sessions consisting of treadmill exercises (30 min) and bicycle exercises (30 min) for a total duration of 1 hour, 3 days per week, for 12 weeks. All participants then underwent 12 weeks of consecutive detraining. To achieve the study objectives, all dependent parameters such as dietary intake, daily activity, body composition, aerobic exercise capacity, and stress-related variables were measured before training, after 12 weeks of training, and after 12 weeks of detraining. Additionally, participants in both groups were instructed to voluntarily take 70% of each individual's recommended daily allowance (RDA) through consultation with a clinical nutritionist during the study period.

### Measurements

#### Dietary treatment and dietary intake

A clinical nutritionist prescribed the appropriate amount needed to lose weight based on age, height, weight, and activity. Dietary intake approximated 70% of the RDA for normal adults^10^. Participants were requested to record details of their meals (i.e., type, amount, and ingredients) every day both before and during the 24-week intervention period, which was monitored by a clinical nutritionist every month to confirm adherence to the dietary treatment. Total caloric intake was analyzed by selecting the 2 times per weekday and 1 time per weekend records at various time points throughout the study. Total caloric intake was analyzed using a Computer Applied Nutrition Analysis Program, according to Korea Nutrition Facts from the Korea Food & Drug Administration in the Ministry for Health, Welfare, and Family Affairs, Republic of Korea.

#### Daily activity

Fitness bands (Polar Loop 1, Finland) were used to examine daily activity in addition to the defined exercise training. The instrument was used to measure the estimated number of calories consumed daily during our study periods.

#### Height and body composition

Height was measured as the distance between the bottom of the foot and top of the head using a stadiometer (PKS-1008, Tokyo, Japan). Body weight, percentage body fat, and BMI were measured using an X-SCANPLUS (Jawon Medical, Seoul, Korea).

#### Aerobic performance

Aerobic performance was analyzed by GXT using K4B2 (Cosmed, Rome, Italy), an automatic breathing gas analyzer. The GXT protocol was started at 3.6 km/h, and while the speed was increased by 1.2 km/h every 2 min, the slope was 0% at all speeds. We finished the exercise test when three or more of the following occurred: (i) when the subject requested to stop the test; (ii) when the current heart rate exceeded 90% of the predicted maximal heart rate; (iii) when the respiratory exchange rate was >1.15; (iv) when the oxygen intake did not change even when the exercise intensity increased. Blood lactate levels were measured every minute by using a lactic acid analyzer (YSI-1500) for blood samples collected into capillary tubes with the finger-tip method.

#### Stress related parameters

The measurement of stress-related parameters were performed using the HRV measuring device (LAXTHA, CANS-3000, Seoul, Korea). The electrocardiogram was measured for 5 minutes, and the data were automatically analyzed using the Telecan version 3.23 program. HRV was analyzed using time-domain methods based on beat-to-beat or normal-to-normal (NN) intervals, and frequency-domain methods assigned to bands of frequency followed by counting the number of NN intervals matching each band. The average of all RR intervals (mean RR), standard deviation of successive differences (SDDN), and root mean squares of successive differences (RMSSN) were analyzed using time-domain methods. Total power (TP), low frequency (LF), and high frequency (HF) were measured as frequency-domain methods.

#### Exercise training

Exercise training during the intervention period was performed as follows: during the treadmill exercises, the heart rates (HR) of all participants were monitored using a HR monitor (Polar S610i, Finland) to maintain their target HR corresponding to LT and LT + 5% as much as possible, and exercise was performed for 30 minutes while controlling the speed of the treadmill (Taeha IT-6025, Korea). For bicycle exercises, all obese women entered their gender, age, weight, HRLT and HRLT + 5% into a cycle ergometer (Combi 75XLII, Tokyo, Japan) and attached a heart rate sensor to the earlobe. The exercise load of the bicycle ergometer was automatically adjusted according to the heart rate information transmitted through the heart rate sensor attached to the earlobe.

### Statistical analysis

Means and standard deviations (SD) were calculated for each primary dependent variable. Normality of distribution of all outcome variables were verified using the Kolmogorov-Smirnov test. A two-way analysis (time × group) of covariance with repeated measures of the “time” factor was used to analyze the effects of training programs on each dependent variable. Post-hoc tests between times in each group, and paired t-tests and post-hoc test between groups at each time point for least significant difference (LSD) were conducted. All analyses were performed using the IBM Statistical Package for the Social Sciences for Windows, version 23 (IBM Corp., Armonk, NY, USA). The level of significance was set a priori at 0.05.

## RESULTS

### Dietary intake

Before, after training, and after detraining data for dietary intake are shown in Table 2. Dietary intake showed no significant interaction and main effect. This means that changes in dietary intake do not affect training and detraining. However, the HR_LT_ and HR_LT_ + 5% exercise groups showed 830.8 kcal and 782.7 kcal dietary intake reduction, respectively, by training. Also, detraining showed a tendency for dietary intake to increase corresponding to 342.8 kcal in the HR_LT_ exercise group and 484.6 kcal in the HR_LT_ + 5% exercise group.

**Table 2.** Changes of dietary intake (kcal/day) during training and detraining periods in each group

Groups	Training at 0 weeks	Training at 12 weeks	Detraining at 12 weeks	F-value
HR_L__T_	2013.06 ± 382.93	1182.22 ± 327.01	1525.01 ± 334.97	T: 3.331 G: .053 T x G: .446
HR_L__T_ + 5%	1868.53 ± 395.37	1085.79 ± 123.47	1570.34 ± 508.23	

Note: HR_L__T_ = heart rate at lactate threshold, T = time, G = group.

### Daily activity

Table 3 depicts before, after training, and after detraining data for daily activity in both groups. There was no significant interaction, but significant main effects within the time periods have been presented. As a result of post-hoc analysis, the HR_LT_ and HR_LT_ + 5% exercise groups showed 221.5 kcal and 284.9 kcal daily activity increase over 12 weeks of exercise training, respectively. However, both the HR_LT_ and HR_LT_ + 5% exercise groups showed 92.3 kcal and 136.9 kcal daily activity reductions by detraining, respectively.

**Table 3.** Changes in daily activity (kcal/day) during training and detraining periods in each group

Groups	Training at 0 weeks	Training at 12 weeks	Detraining at 12 weeks	F-value
HR_LT_	1970.42 ± 187.00	2191.92 ± 181.05^a^	2099.59 ± 107.31^a^^b^	T: 12.650^*^ G: 2.368 TxG: 1.660
HR_LT_ + 5%	2051.96 ± 188.27	2336.95 ± 162.93^a^	2200.04 ± 205.78^a^^b^	

Note: HR_LT_ = heart rate at lactate threshold, T = time, G = group.

* significant interaction or main effect, ^a^ *p*<.05 vs training at 0 weeks, ^b^ *p*<.05 vs training at 12 weeks.

### Body composition

The change in body composition by training and detraining are shown in Table 4. There were no significant interactions in any of the body composition parameters, but significant main effects within time presented in % body fat by detraining. As a result of post-hoc analysis, there were no statistical differences in body weight, but the HR_LT_ and HR_LT_ + 5% exercise groups showed 4.9 kg and 4.8 kg body weight decrease tendencies, respectively, over 12 weeks of training, and this decrease tended to persist after 12 weeks of detraining. Body fat percentage also showed a 3.1% tendency to increase in the HR_LT_ exercise group and 3% in the HR_LT_ + 5% exercise group, but it did not change in either group by 12 weeks of detraining.

**Table 4.** Changes of body composition during training and detraining periods in each group

	Items	Groups	0 weeks	4 weeks	8 weeks	12 weeks	F-value
Training	Weight (kg)	HR_LT_	69.14 ± 7.84	67.18 ± 7.77	65.16 ± 8.65	64.15 ± 8.70	T: .832 G: .009 TxG: .052
		HR_LT_ + 5%	71.20 ± 5.44	69.19 ± 6.40	67.54 ± 5.99	66.35 ± 6.01	
	%BF	HR_LT_	34.23 ± 3.83	32.63 ± 3.92	32.37 ± 3.21	31.14 ± 3.64	T: .908 G: .002 TxG: .297
		HR_LT_ + 5%	34.20 ± 2.01	32.84 ± 2.47	31.90 ± 2.88	31.20 ± 3.07	
	BMI (kg/m^2^)	HR_LT_	27.53 ± 2.54	26.75 ± 2.49	25.94 ± 2.91	25.41 ± 3.22	T: 1.367 G: .069 TxG: .132
		HR_LT_ + 5%	27.26 ± 1.58	26.48 ± 1.97	25.85 ± 1.80	25.22 ± 1.84	
Detraining	Weight (kg)	HR_LT_	64.15 ± 8.70	63.98 ± 9.18	64.06 ± 8.95	63.93 ± 8.68	T: .496 G: .895 TxG: .609
		HR_LT_ + 5%	66.35 ± 6.01	66.79 ± 6.25	66.58 ± 6.44	66.77 ± 6.61	
	%BF	HR_LT_	31.14 ± 3.64	30.35 ± 2.71	31.13 ± 3.02^b^	31.41 ± 3.90	T: 3.863^*^ G: 1.372 TxG: .723
		HR_LT_ + 5%	31.20 ± 3.07	31.11 ± 2.92	31.32 ± 2.76	31.99 ± 3.08^b^	
	BMI (kg/m^2^)	HR_LT_	25.41 ± 3.22	25.33 ± 3.35	25.37 ± 3.30	25.32 ± 3.18	T: .308 G: 1.287 TxG: .766
		HR_LT_ + 5%	25.22 ± 1.84	25.38 ± 1.93	25.30 ± 1.98	25.38 ± 2.07	

Note: % BF = percent of body fat, BMI = body mass index, HR_LT_ = heart rate at lactate threshold, T = time, G = group.

* significant interaction or main effect, ^b^ *p*<.05 vs training at 4 weeks

### Aerobic performance

Table 5 depicts before, after training, and after detraining data for aerobic performance parameters in both groups. There were no significant interactions in any of the` body composition parameters, but significant main effects within time presented in HR_LT_, HR_max_, and VO_2LT_ by training and detraining. As a result of post-hoc analysis, HR_LT_ showed no significant changes in training in either group, but it showed significant decreases of 11.3 bpm and 7.4 bpm in the HR_LT_ and HR_LT_ + 5% exercise groups, respectively, by detraining. HR_max_ decreased significantly with time regardless of the 12 weeks of training and detraining periods. VO_2LT_ showed significant increases of 4.85 mL/kg/min and 4.93 mL/kg/ min by 12 weeks of training in the HR_LT_ and HR_LT_ + 5% exercise groups, respectively. However, VO_2LT_ decreased significantly by 3.8 mL/kg/min and 3.4 mL/kg/min, respectively, after 12 weeks of detraining. In VO_2max_, the HR_LT_ and HR_LT_ + 5% exercise groups showed increase tendencies of 4.86 mL/kg/min and 4.15 mL/kg/min, respectively, by training. Also, VO_2max_ showed decrease tendencies of 4.65 mL/kg/min in the HR_LT_ exercise group and 3.64 mL/kg/min in the HR_LT_ + 5% exercise group.

**Table 5.** Changes in aerobic performance during training in each group

Items	Groups	Training 0 week	Training 12 week	Detraining 12 week	F-value
HR_LT_ (bpm)	HR_LT_	134.43 ± 14.01	142.14 ± 13.29	130.81 ± 9.35b	T: 14.286^*^ G: .157 TxG: .479
	HR_LT_ + 5%	134.25 ± 15.79	138.71 ± 10.24	131.35 ± 13.06^b^	
HR_max_ (bpm)	HR_LT_	188.92 ± 10.28	187.23 ± 10.27	184.15 ± 8.83^a^	T: 3.533^*^ G: .734 TxG: 2.230
	HR_LT_ + 5%	186.48 ± 10.48	181.15 ± 9.33^a^	183.20 ± 9.22	
VO_2LT_ (mL/kg/min)	HR_LT_	24.00 ± 4.48	28.85 ± 3.72^a^	25.05 ± 3.75^b^	T: 20.045^*^ G: .003 TxG: .015
	HR_LT_ + 5%	23.56 ± 4.35	28.49 ± 3.82^a^	25.07 ± 2.48^b^	
VO_2max_ (mL/kg/min)	HR_LT_	37.31 ± 4.09	42.17 ± 5.25	37.52 ± 4.67	T: .382 G: .078 TxG: .195
	HR_LT_ + 5%	35.58 ± 3.08	39.73 ± 4.74	36.09 ± 4.89	

Note: HR_LT_ = heart rate at lactate threshold. HR_max_ = maximal heart rate, VO_2LT_ = oxygen uptake at lactate threshold, VO_2max_ = maximal oxygen uptake, T = time, G = group.

* significant interaction or main effect, ^a^ *p*<.05 vs training at 0 weeks, ^b^ *p*<.05 vs training at 12 weeks.

### Stress related parameters

The changes in stress-related parameters using HRV by training and detraining are shown in Table 6. There were no significant interactions in any of the stress-related parameters, but significant main effects within time presented by 12 weeks of training in mean RR, SDDN, RMSSD, TP, LF, and HF. Also, 12 weeks of detraining resulted in significant main effects in mean RR and HF. As a result of post-hoc analysis, no significant changes in the HR_LT_ exercise group was seen after 12 weeks of training, but the HR_LT_ + 5% exercise group showed a significant increase, especially in mean RR and RMSSD. Twelve weeks of detraining did not yield any significant change in any of the HRV parameters.

**Table 6.** Changes in HRV as stress related parameters within training and detraining periods in each group

	Items	Groups	0 weeks	4 weeks	8 weeks	12 weeks	F-value
Training	Mean RR (ms)	HR_LT_	842.39 ± 122.26	805.31 ± 103.28	824.68 ± 85.55	828.22 ± 83.26	T: 10.504^*^ G: .579 TxG: .720
		HR_LT_ + 5%	815.24 ± 84.30	794.85 ± 86.48	835.83 ± 85.37^b^	854.01 ± 102.54^b^	
	SDNN (ms)	HR_LT_	37.75 ± 16.58	33.43 ± 9.03	35.30 ± 9.68	34.33 ± 6.26	T: 15.704^*^ G: 1.068 TxG: 1.324
		HR_LT_ + 5%	35.88 ± 11.12	34.98 ± 9.40	34.79 ± 9.15	37.90 ± 10.12c	
	RMSSD (ms)	HR_LT_	22.62 ± 15.98	20.24 ± 10.76	21.08 ± 8.38	21.84 ± 7.80	T: 14.094^*^ G: 1.623 TxG: 1.035
		HR_LT_ + 5%	17.93 ± 6.71	20.34 ± 9.02	22.59 ± 9.49^a^	23.80 ± 9.09^a^^b^	
	TP (ms^2^)	HR_LT_	7.01 ± 0.77	6.78 ± 0.56	6.88 ± 0.57	6.87 ± 0.36	T: 7.658^*^ G: .526 TxG: .701
		HR_LT_ + 5%	7.01 ± 0.77	6.86 ± 0.59	6.89 ± 0.52	7.05 ± 0.52^c^	
	LF (ms^2^)	HR_LT_	5.37 ± 0.83	5.25 ± 0.61	5.39 ± 0.46	5.30 ± 0.50	T: 8.583^*^ G: .430 TxG: .838
		HR_LT_ + 5%	5.42 ± 0.68	5.44 ± 0.61	5.39 ± 0.72	5.54 ± 0.72^c^	
	HF (ms^2^)	HR_LT_	5.52 ± 1.40	5.08 ± 1.08	5.08 ± 1.00	5.13 ± 0.82	T: 10.885^*^ G: 1.430 TxG: .834
		HR_LT_ + 5%	5.15 ± 0.88	5.06 ± 0.94	5.16 ± 0.73	5.27 ± 0.81	
Detraining	Mean RR (ms)	HR_LT_	828.22 ± 83.26	839.54 ± 65.77	818.68 ± 82.55	820.12 ± 115.29	T: 3.265^*^ G: .547 TxG: .301
		HR_LT_ + 5%	854.01 ± 102.54	876.39 ± 92.60	847.69 ± 94.55^b^	858.89 ± 98.82	
	SDNN (ms)	HR_LT_	34.33 ± 6.26	36.59 ± 9.80	34.38 ± 10.05	32.31 ± 9.21	T: .536 G: .509 TxG: .551
		HR_LT_ + 5%	37.90 ± 10.12	40.94 ± 12.52	38.73 ± 11.25	38.67 ± 10.54	
	RMSSD (ms)	HR_LT_	21.84 ± 7.80	22.63 ± 9.28	22.15 ± 9.22	19.65 ± 7.60	T: 1.832 G: 1.674 TxG: 1.033
		HR_LT_ + 5%	23.80 ± 9.09	26.60 ± 12.58	24.89 ± 11.54	25.07 ± 10.07	
	TP (ms^2^)	HR_LT_	6.87 ± 0.36	6.92 ± 0.60	6.78 ± 0.60	6.69 ± 0.62	T: 1.206 G: .666 TxG: .960
		HR_LT_ + 5%	7.05 ± 0.52	7.16 ± 0.61	7.02 ± 0.58	7.07 ± 0.49	
	LF (ms^2^)	HR_LT_	5.30 ± 0.50	5.37 ± 0.48	5.20 ± 0.68	5.00 ± 0.71^b^	T: 1.381 G: 1.073 TxG: 1.639
		HR_LT_ + 5%	5.54 ± 0.72	5.59 ± 0.73	5.50 ± 0.62	5.47 ± 0.52	
	HF (ms^2^)	HR_LT_	5.13 ± 0.82	5.09 ± 0.95	5.10 ± 1.05	5.00 ± 0.57	T: 4.966^*^ G: 2.211 TxG: 1.106
		HR_LT_ + 5%	5.27 ± 0.81	5.41 ± 0.86	5.32 ± 0.85	5.40 ± 0.74	

Note. HRV = heart rate variability, SDNN = standard deviation of NN intervals, RMSSD = root mean square of successive differences, TP = total power, LF = low frequency, HF = high frequency, HR_LT_ = heart rate at lactate threshold, T = time, G = group.

* significant interaction or main effect, ^a^ *p*<.05 vs training at 0 weeks, ^b^ *p*<.05 vs training at 12 weeks, # p<.05 vs HR_LT_ group

## DISCUSSION

Our study examined the effect of diet plus exercise training at LT and detraining for 12 weeks on body composition, aerobic performance, and stress-related variables after classifying obese women in their 20s to 40s into HR_LT_ and HR_LT_ + 5% exercise groups.

With regard to dietary intake and daily activity, all participants were encouraged to adopt 70% of the RDA during the 12-week training period. The total calorie intake was significantly decreased during the 12 weeks of exercise training in both groups, and the decrease ratio was found to be 830.8 kcal vs 782.7 kcal (HR_LT_ vs HR_LT_ exercise groups), indicating that the treatment for diet was relatively well done. Daily activity showed a similar increase of 221.5 kcal and 284.9 kcal in the HR_LT_ and HR_LT_ exercise groups, respectively, during the training period. These results suggest that exercise training is a great motivator for weight loss and health management in the participants, and this motivation changed their lifestyle more actively and positively during the training period^21^. However, 12 weeks of detraining resulted in a significant increase in dietary intake and a significant decrease in daily activity in both groups. Therefore, the detraining in obese women in their 20s to 40s is thought to be the result of both a cessation in physical exercise and an induction of the yo-yo phenomenon of weight gain through increasing dietary intake and decreasing daily activity^22^.

The most commonly used method of treating obesity is a combination of diet and exercise, and it has been reported that dietary treatment should not be limited to less than 1,200-1,600 kcal/day for men and 1,000-1,200 kcal/day for women to avoid limiting the energy balance and micronutrient intake required by individuals^23^. Additionally, it is widely known that aerobic exercise of 60 to 90 minutes or more daily is the most commonly used method of treating obesity as it reduces body fat^24^. The combination of diet and exercise has been reported to not only reduce body weight and body fat, but also to improve various clinical factors associated with diabetes and cardiovascular disease^8^^,^^25^. It also increases endorphin secretion to make the subject feel good and has a motivating effect on the treatment of obesity, which can be maintained for a relatively long time after treatment^26^.

Based on this previous study, our study provided proper diet and exercise treatment to all participants of the HR_LT_ and HR_LT_ + 5% exercise groups during the 12-week training period. As a result, body weight showed a decrease of 4.9 kg and 4.8 kg in the HR_LT_ and HR_LT_ + 5% exercise groups, respectively, which persisted after 12 weeks of detraining. The percentage of body fat decreased by 3.1% and 3% in the HR_LT_ and HR_LT_ + 5% exercise groups, respectively, but showed slightly increased tendencies of 0.3% and 0.8% in the HR_LT_ and HR_LT_ + 5% exercise groups, respectively, after 12 weeks of detraining. In other words, our study showed a positive effect contrary to the results of previous studies, in which obese people stopped the long-term diet plus exercise intervention for weight loss and showed the yo-yo phenomenon. The differences compared to previous studies cannot be clearly interpreted, but these results are believed to be due to the fact that although both groups had higher dietary intake and lower daily activity after 12 weeks of detraining than after 12 weeks of training, there was a relatively lower dietary intake and higher daily activity after 12 weeks of detraining than before training.

Generally, the improvement of aerobic performance by long-term exercise training has been reported to improve body composition and various clinically-related variables, thereby reducing the risk of obesity and metabolic syndrome^27^^,^^28^. The most commonly used parameters in evaluating aerobic performance are VO_2max_ and HR_max_. VO_2LT_ and HR_LT_ have also been reported to be appropriate as evaluation parameters^29^^,^^30^. Our study showed a significant increase in VO_2LT_ and increased tendency in VO_2max_ by 12 weeks of exercise training in both groups. However, despite a higher daily activity being maintained during the detraining period than before training, the aerobic performance was reduced to the initial level. These results suggest that aerobic performance is not maintained even if high daily activity is shown through various activities in daily life, unless physical activity of high intensity is performed.

The HRV test is recognized as a very effective method for determining stress levels by quantitatively evaluating the activity and balance of the autonomic nervous system, and the result is a relatively simple non-invasive measurement that can be quickly obtained through computer analysis^30^^,^^31^. Furthermore, HRV has the advantage of being able to objectively and easily measure the activity of the autonomic nervous system and changes in its activity and balance due to stress have been reported to have characteristics that make it possible to diagnose the degree of stress and stress-related diseases in the field of psychiatry^14^^,^^32^. Therefore, the HRV test is regarded as a useful tool for objectively evaluating the psychological-emotional status of an individual^33^. 

In this study, the HRV test was performed to examine the effects of 12 weeks of training and detraining on the stress in obese women in their 20s to 40s. No significant change was observed in the HR_LT_ exercise group, but the HR_LT_ + 5% exercise group showed a relatively marked increase tendency. In particular, mean RR and RMSSD showed significant increases of 38.77 ms and 5.87 ms, respectively. Mean RR and RMSSD, which are prominent in exercise training, are very simple methods that use the RR intervals obtained from electrocardiograms^34^. The RR interval is the interval between R and the next consecutive R between adjacent QRS complexes on the ECG, and mean RR generally corresponds to the mean value of the time interval between R and R for 5 minutes. The RMSSD is expressed as the square root of the average of the sums of squares of differences between adjacent RR intervals. These variables are reported to indicate short-term cardiac variability and activity of parasympathetic nerves. Therefore, it is reported that the autonomic nervous system activity and balance are stable, less stressed, and healthy people have higher mean RR and RMSSD levels^14^^,^^17^. Also, these HRV variables have been reported to improve with various indicators for obesity and lifestyle diseases when applying various types of exercise training^14^^,^^17^^,^^30^^,^^33^. Therefore, dietary treatment equivalent to 70% of RDA and exercise treatment above moderate intensity corresponding to HR_LT_ and HR_LT_ + 5% for 12 weeks enhance the balance of the autonomic nervous system and resistance to stress by improving HRV, and these effects are believed to persist even after 12 weeks of detraining.

Our results suggest that 70% RDA of dietary intervention and exercise training corresponding to HR_LT_ and HR_LT_ + 5% for 12 weeks were effective in improving body composition, aerobic performance, and stress. In particular, the improvement of HRV, an indicator of stress, persisted for up to 12 weeks after the end of exercise training in obese women.
